# Climate-influenced vector-borne diseases in Africa: a call to empower the next generation of African researchers for sustainable solutions

**DOI:** 10.1186/s40249-024-01193-5

**Published:** 2024-03-14

**Authors:** Judicaël Obame-Nkoghe, Adjoavi Esse Agossou, Gerald Mboowa, Basile Kamgang, Cyril Caminade, Dawn C. Duke, Andrew Karanja Githeko, Obed M. Ogega, Nestor Engone Elloué, Fatou Bintou Sarr, Dieudonné Nkoghe, Pierre Kengne, Nicaise T. Ndam, Christophe Paupy, Moses Bockarie, Patricks Voua Otomo

**Affiliations:** 1 Molecular and Cellular Biology Laboratory (LabMC), Biology Department, University of Science and Technology of Masuku, BP 901, Franceville, Gabon; 2 Ecology and Health Research Unit, Interdisciplinary Center for Medical Research of Franceville, BP 769, Franceville, Gabon; 3 Department of Zoology and Entomology, Faculty of Natural and Agricultural Sciences, University of the Free State, Private Bag x13, Phuthaditjhaba, 9866 Republic of South Africa; 4 Laboratory of Pharmacology and Improved Traditional Medicines, Department of Animal Physiology, Faculty of Science and Technology, University of Abomey-Calavi, BP 526, Cotonou, Benin; 5 The African Center of Excellence in Bioinformatics and Data-Intensive Sciences, Infectious Diseases Institute, College of Health Sciences, Makerere University, P. O Box 22418, Kampala, Uganda; 6 Africa Centers for Disease Control and Prevention, African Union Commission, Roosevelt Street, P.O. Box 3243, W21 K19 Addis Ababa, Ethiopia; 7 Centre for Research in Infectious Diseases, P.O. Box 13591, Yaoundé, Cameroon; 8 Earth System Physics Department, The Abdus Salam International Centre for Theoretical Physics, Trieste, Italy; 9 Africa Research Excellence Fund, 99 Charterhouse Street, London, EC1M 6HR UK; 10 Community Health Support Program, P.O. Box 872, Kisumu, 40100 Kenya; 11 The African Academy of Sciences, Nairobi, Kenya; 12 Center for Phylosophical Studies and Research (CERP), Omar Bongo University (UOB), BP 13131, Libreville, Gabon; 13 UMRED, Health Training and Research Unit, University of Iba Der Thiam of Thiès, BP 967, Thiès, Senegal; 14 National Parasitic Diseases Control Program, Ministry of Health, Libreville, Gabon; 15 MIVEGEC, Univ. Montpellier, CNRS, IRD, Montpellier, France; 16 MERIT, IRD, Paris Cité University, 75006 Paris, France; 17 Department of Parasitology, Noguchi Memorial Institute for Medical Research, University of Ghana, LG 54, Accra, Ghana; 18 School of Community Health Sciences, Njala University, Bo, Sierra Leone

**Keywords:** Climate change, Vector-borne diseases, Africa, Environmental justice, Research and development, Pan-African funding initiatives

## Abstract

We look at the link between climate change and vector-borne diseases in low- and middle-income countries in Africa. The large endemicity and escalating threat of diseases such as malaria and arboviral diseases, intensified by climate change, disproportionately affects vulnerable communities globally. We highlight the urgency of prioritizing research and development, advocating for robust scientific inquiry to promote adaptation strategies, and the vital role that the next generation of African research leaders will play in addressing these challenges. Despite significant challenges such as funding shortages within countries, various pan-African-oriented funding bodies such as the African Academy of Sciences, the Africa Research Excellence Fund, the Wellcome Trust, the U.S. National Institutes of Health, and the Bill and Melinda Gates Foundation as well as initiatives such as the African Research Initiative for Scientific Excellence and the Pan-African Mosquito Control Association, have empowered (or are empowering) these researchers by supporting capacity building activities, including continental and global networking, skill development, mentoring, and African-led research. This article underscores the urgency of increased national investment in research, proposing the establishment of research government agencies to drive evidence-based interventions. Collaboration between governments and scientific communities, sustained by pan-African funding bodies, is crucial. Through these efforts, African nations are likely to enhance the resilience and adaptive capacity of their systems and communities by navigating these challenges effectively, fostering scientific excellence and implementing transformative solutions against climate-sensitive vector-borne diseases.

## Background

Climate change-driven spread and transmission intensification of vector-borne diseases are some of the most pressing global health and environmental issues [1]. This is even more true in Africa's low- and middle-income countries (LMICs) where social, economic, and health disparities are at the core of recurring public health crises [2, 3]. Due to global warming, largely driven by anthropogenic greenhouse gas emissions and deforestation, prediction of climatic patterns is becoming increasingly difficult [4], especially in the LMICs where resources are limited [5–7]. These environmental shifts can impact the proliferation and seasonality of vector-borne diseases such as malaria, dengue, chikungunya, Rift Valley fever, Zika, and other diseases [8, 9]. Field and modelling studies highlighted that *Anopheles* species have already moved to higher elevation and latitudes [10]. The length of the malaria transmission season and population at risk could increase over highland regions of Africa, while they could decrease over the warmest plains in future [11]. Hence, robust scientific inquiry is essential in understanding the complex dynamics of climate change's impact on disease occurrence and transmission patterns and their effect on vulnerable communities and systems.

Research not only provides crucial insights into the changing environment landscape but also informs adaptive strategies tailored to unique needs such as vector control, chemotherapy, and immunization interventions in LMICs. Yet, most African governments in LMICs still struggle to support such initiatives effectively and perennially, and the lack of sufficient trained human capital to tackle this issue only exacerbates the problem. Faced with these challenges, the need for a critical mass of leaders capable of conceptualizing and implementing innovative and sustainable strategies to mitigate the impact of vector-borne diseases intensified by climate change in Africa is more than pivotal. In the midst of these intricacies, emerging African research leaders must enter the field as catalysts of change, gifted with an intercultural fluency that arms them with a keen understanding of local dynamics, national and cross-boundary complexities, along with the ability to work across disciplines to lead innovation and reshape the narrative.

**Fig. 1:**
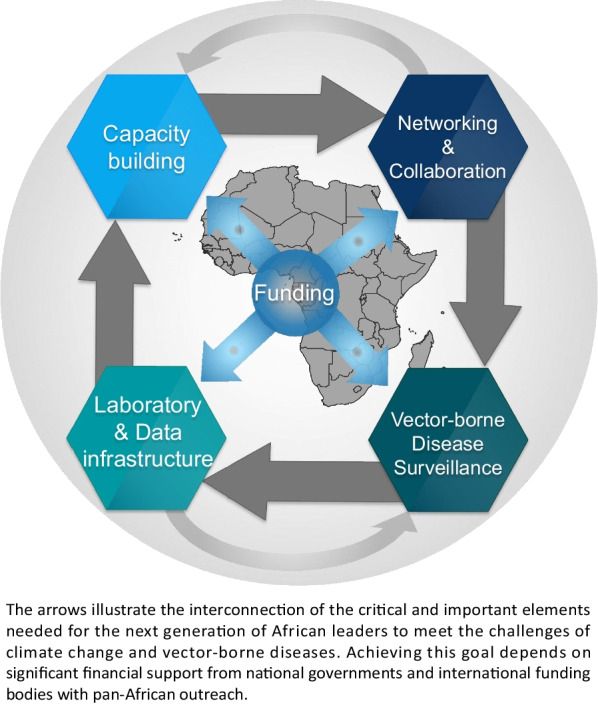
Critical components in building the next generation of African researchers working on vector-borne diseases

In this article, we highlight the vital role that the next generation of African research leaders must play in addressing the complex challenges posed by climate change and vector-borne diseases within African LMICs. More specifically, we first elucidate the challenges faced by African LMICs in dealing with these interrelated issues and emphasize why the engagement of emerging scholars is of paramount importance. Then, we underscore the distinctive strengths and contributions that the next generation of African research leaders are bringing to the table in addressing the intricate intersection of climate change and vector-borne diseases. Finally, we advocate for joint efforts, including increased investment of African LMIC governments in research capacity and programs and the strengthening of regional and continental research networks that will help set the agenda while shaping a resilient and sustainable future for African LMICs (Fig. 1).

## Main text

### Information searching strategy

**Fig. 2:**
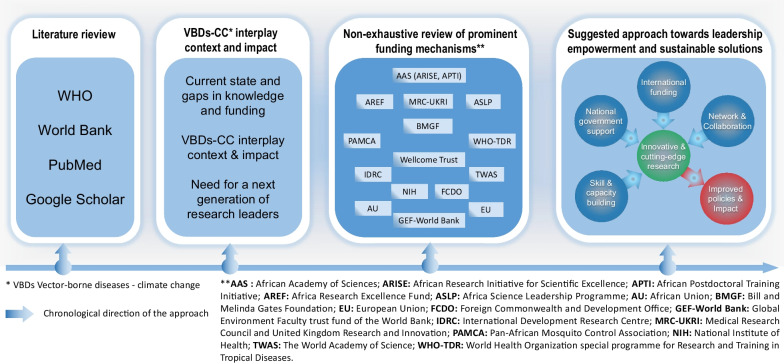
Chronological frame towards sustainable solutions and impact in response to climate change challenges in Africa

To elucidate the intricate nexus between climate change, vector-borne diseases, and the imperative role of emerging research leaders in LMICs within the African context, we adopted a meticulous information research strategy to ensure a comprehensive exploration of the existing literature before organizing retrieved information and suggest approach towards leadership empowerment and sustainable solutions (Fig. 2). For that, we undertook a literature review encompassing peer-reviewed articles and reports from reputable international health organizations (e.g., The World Health Organization or the World Bank). Using various combinations of keywords including “climate change”, “vector-borne diseases”, “Africa”, “research and development”, “environmental justice”, and “funding initiatives”, we queried PubMed (https://pubmed.ncbi.nlm.nih.gov) and Google Scholar (https://scholar.google.com) databases for articles’ search, ensuring a thorough retrieval of scholarly information on the subject matter.

Additionally, a meticulous examination of funding mechanisms supporting research initiatives in LMICs in Africa was conducted. This involved a search in Google (https://www.google.com/) and an in-depth but non-exhaustive analysis of initiatives with a Pan-African outreach providing substantial funding to support vector-borne diseases-based research and the empowerment provided to the next generation of African researchers, particularly in the realms of skill development, mentoring, and research capacity building. Key insights into the activities and the type of support provided by the funding bodies or initiatives inventoried were summarized based on consulting their respective websites.

### Setting the stage: climate change, vector-borne diseases, and their growing impact on African LMICs

Vector-borne diseases account for more than 17% of all infectious diseases, causing more than 700,000 deaths annually [12]. At the global level, malaria (219 million cases, 400,000 deaths every year) and dengue (3.9 billion people at risk, 96 million symptomatic cases, 40,000 deaths every year) are associated with the largest burden [12]. Other major threatening vector-borne diseases, including but not limited to yellow fever, chikungunya, Zika virus fever, West Nile fever, Japanese encephalitis, Chagas diseases, and leishmaniasis, affect hundreds of millions of people worldwide [12]. Arboviruses have become important and constant threats in tropical regions due to rapid climate change, environmental change (deforestation, farming, mining, etc.), population migration, uncontrolled urbanization, and precarious sanitary conditions that favor viral amplification and transmission [13, 14].

In the field of global health and environmental challenges, few issues rival the worrying convergence of climate change and vector-borne diseases. As the world grapples with many facets of a changing climate, it is imperative to recognize that its consequences are not evenly distributed. African LMICs, already facing a myriad of socioeconomic and health disparities, find themselves at the forefront of this climate-induced battle against the growing threat of vector-borne diseases. The scientific consensus is unequivocal: our planet is undergoing a period of unprecedented warming, driven primarily by human activities [15]. This inexorable rise in global temperatures has triggered an era of climate uncertainty, characterized by unpredictable rainfall patterns, prolonged droughts, and intensified extreme weather events [15]. Associated with socioeconomic vulnerabilities, these environmental shifts have primed African LMICs to bear the burden of the deleterious health consequences of climate change. Central to this impending crisis is the increase in temperature, creating breeding grounds for disease vectors and lengthening transmission seasons [16]. Many *Anopheles* vectors of malaria and *Aedes* vectors of arboviruses have developed resistance to insecticides, and alternative vector control solutions such as the sterile insect technique (SIT) or genetically modified mosquitoes are slow to become operational [17] and sometimes costly, resulting in an additional layer of complexity. Warmer temperatures are also associated with increasing biting rates, promoting the vectorial capacities of arthropod vectors [18, 19], and speeding up the development of pathogens within vectors, reducing the extrinsic incubation period and thus the replication rate (*R*_0_) of infection in human populations [20]. These changes increase the transmission potential of diseases such as malaria, dengue, or Zika and a panoply of other viral and bacterial infections transmitted by vectors such as mosquitoes, ticks, midges, and flies [8].

Global warming, combined with globalization and the movement of goods and vectors worldwide [21], is redrawing a map of vector-borne diseases. Indeed, as temperatures rise and precipitation patterns shift, the geographic ranges of vectors expand in latitude and altitude, allowing these disease carriers to infiltrate previously unaffected regions [8]. For instance, vectors have been introduced in Europe and in the United States of America (USA) and sometimes from India to Africa (e.g., *An. stephensi*) via the shipment of goods, mostly in containers [21]. Altered climatic conditions modify the temporal and spatial distribution of pathogens [22]. Furthermore, due to climate change-driven uncertainty, in some regions, rainfall can decrease with a dramatic consequence for vector population dynamics (*Anopheles,* for example) and a positive effect for others (such as *Aedes* or *An. stephensi* with the increase in man-made water storage), thus setting the stage for outbreaks in areas often inadequately prepared to face them. Species once confined to specific regions now venture into new territories, exposing previously unaffected and nonimmune populations to diseases that they are not prepared to contain physiologically or logistically [8]. The tropical and subtropical regions of Africa, which are already vulnerable, are witnessing the relentless expansion of vectors such as *Aedes* mosquitoes, which are capable of transmitting diseases such as dengue, yellow fever, chikungunya, and Zika [23]. This geographical shift not only threatens human health but also strains healthcare systems that must cope with the emergence of unfamiliar diseases. It is in these settings where the burden of vector-borne diseases has long exerted a toll, and climate change exacerbates an already daunting challenge. In this respect, the link between climate change and vector-borne diseases is palpable and detrimental, rooted in the complex dynamics of vector ecology, pathogen spread, and human vulnerability [24].

The implications of this climate-driven vector-borne disease convergence are unsettling. Indeed, African LMICs are already struggling with resource limitations, fragile healthcare systems, and socioeconomic inequalities [25]. Another aspect of the climate challenge is that the vast majority of livelihoods within these LMICs remain strongly related to climate-sensitive sectors such as rainfed agriculture [26, 27], where climatic fluctuations can precipitate food insecurity, undernutrition, and a cascade of health challenges [28]. One important challenge is the brain drain issue, observed when excellent African scientists decide to live in Europe, the USA, or other developed countries overseas for economic reasons. These limitations, combined with inadequate access to quality education, result in a lack of highly skilled human resources, making African LMICs extremely vulnerable to the health fallout of this climate-induced threat, which in turn is likely to critically affect their public health resilience efforts [24, 29, 30].

Furthermore, the geographical and climatic diversity of the African continent, highlighted either by retrospective, ongoing, or predictive studies [31–33], introduces an additional layer of complexity. Indeed, the heterogeneity in climate zones, ecosystems, and vector species distribution means that the challenges posed by vector-borne diseases are region-specific and require tailored interventions. For example, the vectors of Rift Valley fever and their ecological preferences vary greatly between West and East Africa [34]. What works in one African LMIC might not necessarily be applicable in another one. This prompts the need for nuanced approaches tailored to local conditions and communities [35]. Consequently, these shortcomings bring vast segments of the population to a state of heightened vulnerability and weigh heavily on the capacity of states to manage and mitigate vector-borne diseases intensified by climate change [28, 29].

The most critical aspect of vulnerability lies in the interplay between environmental and socioeconomic factors. For instance, poor communities often lack the resources for environmental adaptation, preventive healthcare and/or community education and awareness programmes [36]. This amplifies their vulnerability to vector-borne diseases, illustrating how environmental changes driven by climate change disproportionately affect the poorest, further perpetuating socioeconomic and educational disparities and issues of climate justice. A critical examination of these interlinked factors reveals that vulnerability is not merely a result of environmental shifts but is deeply embedded in the socioeconomic context. Thus, any effective strategy to address vulnerability in the context of climate change and vector-borne diseases must encompass not only environmental adaptation but also socioeconomic empowerment. For that, the application of principles of equity in health care and a mitigation framework toward environmental justice can address the constraint imposed by socioeconomic disparities.

The gravity of the current situation calls for a critical examination of the role of the next generation of African research leaders, as human capital assets for the continent’s future, in catalyzing change, aligning research priorities, and mobilizing resources to address this pressing challenge. Therefore, as we delve into the intricate web of climate change’s impact on vector-borne diseases and their consequences for African LMICs, it becomes evident that this is not merely a crisis of health systems, socioeconomic disparities, or environmental justice but an overriding need for skilled human resources to conceive effective and sustainable solutions adapted to Africa’s heterogeneous environmental and socioeconomic contexts. Understanding these dynamics is crucial to developing resilience and effective strategies against climatic threats related to vector-borne diseases. This requires the development of targeted interventions with innovative and evidence-based strategies (e.g., city vegetation, controlled irrigation, and dam building), robust surveillance systems, and multidisciplinary collaborations to significantly reduce the threat of vector-borne diseases due to climate change in African LMICs. In addition, the next generation of researchers must develop networks that enable them to access the often highly competitive funding from major funding organizations. This should complement state funding. However, to sustain such funding support, national governments must support the facilitation of research and ensure that the research addresses critical climate change and health issues.

### The need for a next generation of African research leaders to address climate change and vector-borne disease issues in Africa

The imperative of addressing climate change and vector-borne diseases in Africa requires a focus on nurturing a new generation of research leaders with appropriate skills and understanding of the interaction between climate change and variability in disease transmission and control. These emerging scholars, deeply grounded in their local contexts, possess unique understandings that are essential to meeting the challenges of climate change and vector-borne diseases. In that sense, climate-oriented initiatives to encourage emerging researchers in Africa must be put in place to fill critical gaps in the understanding of health and environmental issues related to climate change and to define mitigation strategies adapted to local contexts.

One way of harnessing the full potential of Africa’s next generation of scientific leaders is by supporting innovative research projects that make a significant contribution to building resilience to environmental threats. The involvement of the scientific leaders should not only enhance scientific knowledge but also lead to practical solutions tailored to African landscapes. These emerging research leaders will need a broader range of transferable and leadership skills, including the ability to work across disciplines and sectors [37]. These are skills that are not traditionally embedded in standard doctoral or postdoctoral training but will be critical to supporting health in this complex and changing environment [38, 39]. By investing in the development and support of this rising generation, African nations can harness the power of local expertise to develop sustainable strategies that ensure the well-being of their people in the face of these challenges.

### Funding shortages: a barrier to progress

Amid the growing challenges posed by vector-borne diseases exacerbated by climate change, a major obstacle has emerged: the inadequacy of publicly funded research initiatives. In-depth understanding and effective mitigation strategies require significant investment in research. The lack of strong government-backed programmes limits the continent’s scientific community and hinders the development of sustainable and well-adapted solutions. This funding gap obstructs progress in key areas of disease prevention and control, limiting Africa’s ability to proactively combat these diseases.

Another face of the problem is the difficulty of obtaining international funding. An analysis by Overland et al. [40] of the dimensions research grant database revealed that from 1990 to 2020, only 3.8% of global funding for climate change research was allocated to African topics. Coussens [41] revealed that the limited research achievement of young African researchers in recent years is linked to inadequate funding, insufficient support to obtain grants, a shortage of training opportunities for skills development, and job instability. Furthermore, research funding from high-income countries is often focused on research agendas created outside of Africa without inputs from researchers and communities from within the countries in which the research is being conducted and is most needed. This leads to a disconnect between the needs of countries and communities and the health research being conducted within them. Furthermore, when funding agendas for Africa are set outside of Africa, there is a reduced ability to adapt research flexibly to meet rapidly changing public health needs driven by climate change [42]. These facts underline the urgent need to increase funding quotas for African researchers and promote African ownership of climate change research for well-adapted and well-informed responses on the ground.

### Current prominent pan-African-oriented funding bodies and initiatives

In their quest to tackle the multifaceted challenges posed by climate change, vector-borne diseases, and socioenvironmental conditions in African LMICs, emerging scientists are finding essential support in a number of continent-wide funding programs. These initiatives, with their pan-African reach, aim to nurture the potential of these young researchers and channel their efforts toward finding innovative solutions to pressing human health issues. Prominent funding bodies providing substantial support for emerging African researchers include but are not limited to organizations or initiatives such as the African Academy of Sciences (AAS) and its programmes including the African Research Initiative for Scientific Excellence (ARISE, https://aasciences.africa/Programmes/ARISE) and the African Postdoctoral Training Initiative (APTI, https://aasciences.africa/Programmes/APTI). Others include the Pan-African Mosquito Control Association (PAMCA, https://pamca.org/en), the International Development Research Centre (IDRC, https://idrc-crdi.ca/en), the National Institute of Health (NIH, https://www.nih.gov), the Global Environment Facility Trust Fund of the World Bank (GEF-World Bank, https://fiftrustee.worldbank.org), the Medical Research Council and UK Research and Innovation (MRC-UKRI, https://www.ukri.org/councils/mrc/), the World Health Organization's Special Programme for Research and Training in Tropical Diseases (WHO-TDR, https://tdr.who.int), the Wellcome Trust (https://wellcome.org), the Bill and Melinda Gates Foundation (BMGF, https://www.gatesfoundation.org), the World Academy of Sciences (TWAS, https://twas.org), the Africa Research Excellence Fund (AREF, https://africaresearchexcellencefund.org.uk), the Foreign, Commonwealth and Development Office (FCDO, https://www.gov.uk/government/organisations/foreign-commonwealth-development-office), and the Africa Science Leadership Programme (ASLP, https://aslp.science). Below are summaries of their respective scopes.

*ARISE*: An innovative scientific exchange initiative implemented by the African Academy of Sciences (AAS) in partnership with the African Union (AU) and the European Union (EU). Launched in December 2020, the current pilot phase supports nearly 600 early- to mid-career researchers across Africa, led by 47 principal investigators (ARISE Fellows) hosted in 38 African countries. The ARISE model is directly inspired by the successful experience of the European Research Council grants initiative—but with an explicit willingness to take into account the specificities of the various African contexts, especially in covering as much as possible all countries in the continent. With five-year research grants of up-to EUR 500,000, ARISE Fellows are supported to implement cutting-edge research projects in various research areas including public health—with a strong emphasis on transdisciplinary research in favor of sustainable development. Anchored on the AU-EU High Level Policy Dialogue on Science, Technology, and Innovation, ARISE fellowships thrive on research and innovation exchanges within Africa and with counterparts in the EU and beyond—using a transdisciplinary approach to address some of the cross-cutting global health issues. By fostering collaborations and offering substantial funding to emerging African researchers, enabling them to act as lead investigators on various research projects, ARISE catalyzes the search for innovative solutions to address cutting-edge research themes, including climate change, vector-borne diseases, and broader health concerns in LMICs.

*APTI*: An initiative of the AAS in partnership with the Bill and Melinda Gates Foundation and the US National Institutes of Health (NIH) that links African early-career researchers (APTI Fellows) with established researchers at various laboratories and centers at the NIH. The APTI Fellows spend two years at the NIH, honing their research skills and trying out their research ideas under the mentorship of senior researchers. Thereafter, the early-career researchers are supported with research grants to return to their home institutions in Africa to implement research projects for another two years. Here, APTI Fellows are supported to produce cutting-edge research while creating and supporting vibrant research ecosystems in their home institutions. Additionally, the fellows are linked to existing regional and global scientific networks for purposes of scientific exchanges and capacity enhancement. Ultimately, the fellows become part of that critical mass of emerging health research leaders that will help deliver a healthy continent.

*AREF*: This organization plays a pivotal role in enhancing research leadership across Africa. Through short fellowship opportunities and professional development programmes, early-career African researchers benefit from mentorship, training, and funding to conduct impactful research on the continent. AREF focuses on supporting talented researchers without prescribing specific research areas and ideas, enabling emerging research leaders to develop their skills and research areas in line with the needs of their context and communities.

*PAMCA*: This organization consists of building capacities for vector-borne disease surveillance and elimination. It aims to promote young researchers involved in vector control-related research and promote collaboration and partnership with key stakeholders in the vector-borne disease elimination agenda. In particular, PAMCA is committed to strengthening the role of women in vector control to achieve gender empowerment, inclusivity, and equity and strengthening governance structures for organizational sustainability.

*IDRC*: The IDRC offers grants, funding, and awards to researchers and institutions to find solutions for global development challenges. More specifically, this organization funds a project that aims to foster various development sectors, including but not limited to climate-resilient food systems, global health, education and science, and sustainable and inclusive economies in developing countries. By promoting gender equity and inclusion as a central strategy in the supported research, the IDRC significantly contributes to fostering the rise of emerging researchers from various layers of society to address climate change-related issues in LMICs.

*NIH*: The NIH offers a range of grant programs aimed at addressing critical global challenges, with a particular focus on climate issues in LMICs. These grants provide essential funding and resources to researchers, organizations, and institutions working, among others, toward climate resilience and sustainability in vulnerable regions. These grants facilitate rigorous, interdisciplinary, and collaborative research and innovative approaches to tackle climate change adaptation.

*GEF-World Bank*: The GEF-World Bank is at the forefront of funding initiatives dedicated to addressing climate issues in LMICs. The GEF provides vital grants that empower LMICs to tackle climate change, enhance environmental sustainability, and promote green growth. Through climate-focused grants, the GEF supports innovative projects that facilitate clean energy adoption, conserve biodiversity, and enhance climate resilience in vulnerable communities. By fostering partnerships between governments, communities, and the private sector, GEF grants enable LMICs to implement transformative solutions, leading to lasting environmental and socioeconomic benefits.

*MRC-UKRI*: The MRC and the UKRI jointly fund research programs that span the African continent. One prominent example is the “African Research Leaders” funding program. These programs provide crucial support for young researchers, enabling them to delve into critical health issues, including those linked to climate change and vector-borne diseases. The funding supports various research areas, from public health to transdisciplinary projects.

*WHO-TDR*: This organization places a significant emphasis on fostering research leadership in LMICs. Their initiatives actively engage young African researchers in cutting-edge research on diseases such as malaria, dengue, and neglected tropical diseases. Through targeted grants and fellowships, the WHO-TDR intends to empower the next generation of African research leaders.

*Wellcome Trust*: The Wellcome Trust is committed to advancing global health, including in Africa. Their funding programs offer opportunities for young African researchers to investigate, among others, the intricate connections between health, climate, and disease vectors. These initiatives support a wide range of research, from basic biology to projects with direct implications for public health.

*BMGF*: Through its diverse funding programs, the foundation empowers emerging scholars in Africa to pursue innovative research in fields crucial to the continent's development, including public health, agriculture, and education. By providing substantial financial support, mentorship, and access to cutting-edge facilities, the foundation makes it possible for several African researchers to drive impactful change through research. A recent BMFG Global Challenges call specifically assigned 80% of the funding to African institutions.

*TWAS*: TWAS is a renowned international organization that focuses on the advancement of science and engineering for sustainable development. Specifically, TWAS is committed to supporting emerging young scientists from developing countries, including African nations, to enhance their research capabilities and contribute significantly to scientific progress. TWAS provides various fellowships, grants, and research opportunities tailored to young scientists, empowering them to pursue innovative research, develop their skills, and collaborate with international peers.

*FCDO*: The FCDO is a department of the United Kingdom government that is responsible for promoting the country's interests overseas, supporting its citizens and businesses around the world, and delivering international development and humanitarian assistance. Regarding funding opportunities for research in Africa, the FCDO, through various initiatives and programs, supports research and development projects in African countries. These initiatives often focus on areas such as health, education, economic development, governance, and environmental sustainability. The FCDO collaborates with research institutions, universities, nongovernmental organizations (NGOs), and other partners to fund projects that aim to address challenges and improve the lives of people in Africa.

*ASLP*: This initiative provides a unique platform for emerging African research leaders to refine their leadership skills. Beyond financial support, this program equips participants with tools to address complex interdisciplinary challenges. It empowers them to lead collaborative research efforts addressing pressing health and environmental issues.

These continental-scale funding organizations or initiatives not only provide essential financial support but also foster a culture of research excellence. They equip emerging African researchers with the skills and resources to meet the challenges posed by climate change, vector-borne diseases, and socioenvironmental implementation conditions in Africa's LMICs.

### The need for greater national investment to support emerging research talents

African countries are at decisive crossroads in the fight against climate change and vector-borne diseases. The urgency of the situation requires substantial investment in research capacity and projects within these nations. Regional and national initiatives tailored to the specific needs and vulnerabilities of each African country are crucial. These initiatives should encompass a spectrum of support measures, from funding for cutting-edge research projects to scholarships fostering the growth of budding scientists. Such investments will enable emerging researchers to explore innovative solutions, including advanced disease surveillance systems [44]. For instance, one key to impact would be to create or enhance government research agencies dedicated to addressing climate-related public health and environmental issues. These agencies can act as catalysts, fostering collaboration between researchers, policymakers, and public health officials. By leveraging the collective knowledge of scientists and the strategic vision of governments, these bodies can drive focused research, expedite data sharing, and facilitate the implementation of evidence-based interventions. To ensure their impact and sustainability, conceptualizing and implementing innovative models of research capacity strengthening targeting indicators at the individual and institutional levels becomes highly necessary [43].

Additionally, such agencies can streamline funding mechanisms, ensuring that research projects align with national priorities. An example of such an agency is the South African National Research Foundation (NRF), which serves as an intermediary between the South African Government and the country’s research institutions (National Research Foundation—South Africa, https://www.nrf.ac.za). The establishment of these collaborative frameworks represents a proactive step toward building resilience and preparedness in the face of climate-induced health challenges, underscoring the vital importance of a coordinated approach to combating vector-borne diseases under the shadow of a changing climate. Furthermore, collaborative networks at the regional level will enhance knowledge exchange, encouraging joint research efforts to combat the unique challenges posed by climate change and vector-borne diseases in each African region [44]. By committing to such investments, African nations will, not only strengthen their research capacities but also foster a culture of scientific excellence, enabling the implementation of transformative solutions for Africa, based on African-led research to tackle the complex interplay of climate change and vector-borne diseases.

## Conclusions

This article highlights critical insights into the intricate interplay between climate change, vector-borne diseases and the role of young African research leaders in mitigating foreseeable effects on human health. First, it highlights the growing threat of vector-borne diseases under the influence of a changing climate, necessitating adaptive strategies. Second, it underscores the unique challenges faced by African LMICs—driven by socioenvironmental determinants. Third, it showcases the emergence of young African research leaders, supported by continental-scale funding programs as potential agents of change. Finally, it advocates for substantial national and regional investments in research capacity and projects, with a distinct focus on tackling vector-borne diseases exacerbated by climate change. The article stresses the importance of strengthening existing collaborative networks and establishing new networks to address urgent health challenges in Africa. The One Health approach, that considers that our health is intrinsically related to health of animals and the environment, is another complementary important approach to address global health problems and meet several sustainable development goals in Africa. In summary, these insights serve as a roadmap for African nations to navigate these complexities and emerge as more influential drivers of change in addressing urgent health and environmental challenges across the continent. In that sense, establishing research bodies with a pan-Africa outreach echoes Colin Carlson’s thought that “an international research institute focusing on the Global Burden of Climate change should be based in countries at the frontlines of climate change impacts. The African continent will seem to be the perfect host for such [an] institute” [45].

## Data Availability

All data generated or analyzed during this study are included in this published article.

## Abbreviations

AAS: African Academy of Sciences
ARISE: African Research Initiative for Scientific Excellence
APTI: African Postdoctoral Training Initiative
AREF: Africa Research Excellence Fund
ASLP: Africa Science Leadership Programme
AU: African Union
BMGF: Bill and Melinda Gates Foundation
EU: European Union
FCDO: Foreign Commonwealth and Development Office
GEF: Global Environment Faculty trust fund
IDRC: International Development Research Centre
LMIC: Low and Middle-Income Country
MRC-UKRI: Medical Research Council and United Kingdom Research and Innovation
NRF: National Research Foundation
NGO: Nongovernmental Organization
PAMCA: Pan-African Mosquito Control Association
NIH: National Institute of Health
SIT: Sterile Insect Technique
R_0_: Replication rate
TWAS: The World Academy of Science
USA: Uniter States of America
WHO-TDR: World Health Organization special programme for Research and Training in Tropical Diseases
